# Application of Mass Spectrometry-Based Metabolomics and Machine Learning in the Diagnostics of Lyme Neuroborreliosis

**DOI:** 10.1021/acsomega.5c10792

**Published:** 2026-03-12

**Authors:** Ilari Kuukkanen, Geraldson Muluh, Đorđe Klisura, Elisa Kortela, Annukka Pietikäinen, Leo Lahti, Jukka Hytönen, Maarit Karonen

**Affiliations:** † Department of Chemistry, 8058 University of Turku, Turku 20014, Finland; ‡ Department of Computing, 8058 University of Turku, Turku 20014, Finland; § 159841 HUS Inflammation Center, Helsinki 00029, Finland; ∥ Institute of Biomedicine, University of Turku, Turku 20014, Finland; ⊥ TYKS Laboratories, Clinical Microbiology, Turku University Hospital, Turku 20014, Finland

## Abstract

Lyme borreliosis (LB) and its disseminated nervous system manifestation, Lyme neuroborreliosis (LNB), presents diagnostic challenges, especially in seropositive and ambiguous clinical cases. In this study, we applied mass spectrometry (MS)-based metabolomics combined with machine learning (ML) to analyze serum samples from patients with definite acute LNB (n = 34), treated LNB (n = 34), together with *Borrelia* antibody-negative (non-LNB) controls (n = 62). Importantly, pre- and post-treatment samples were collected from the same individuals, enabling within-patient comparisons that enhance sensitivity to LNB-related metabolic changes. The non-LNB control group was age- and sex-matched (n = 34), and treated LNB patients served as a practical substitute for postinfectious recovery. Strong discriminatory performance was observed across all pairwise group comparisons. ML model classifiers yielded accuracy rates significantly above those expected by chance, with a perfect classification (1.00) achieved between treated LNB and non-LNB controls. This high separation, independent of antibody status, highlights the potential of MS-based metabolomics as a complementary diagnostic strategy. Receiver operating characteristic curve (ROC) analyses further supported robust performance, with high sensitivity and specificity. Although variance explained in unsupervised ordination was limited (PERMANOVA 4%), the supervised models demonstrated diagnostic value. These findings support the feasibility of metabolomic profiling combined with ML models for LNB diagnosis.

## Graphical abstract

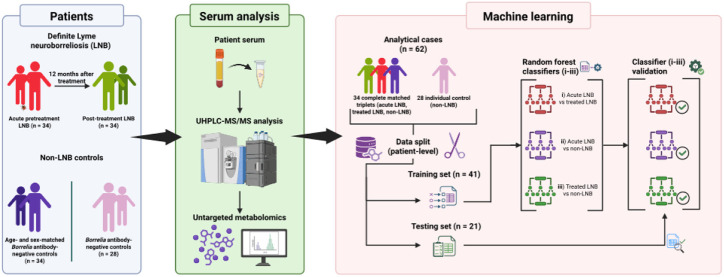

Lyme borreliosis (LB) is caused by the transmission of *Borrelia burgdorferi* sensu lato spirochetes (hereafter referred to as *Borrelia*) through the bites of infected genus *Ixodes* ticks. During the tick’s blood meal, *Borrelia* migrates from the tick midgut to the human skin, leading to localized skin infection. A hallmark of early localized LB is known as erythema migrans (EM), a slowly expanding rash at the feeding site of the tick, but this is not always the case.^1,2^ If untreated at this stage, the infection may progress to early disseminated LB, where *Borrelia* spreads from the initial feeding site via hematogenous or lymphatic routes, potentially affecting multiple organs.^3^ The manifestation of early disseminated LB, which is characterized by *Borrelia* crossing the blood-brain barrier and invading the nervous system and, in some cases, the central nervous system (CNS), is referred to as Lyme neuroborreliosis (LNB).^1,4–7^ The diagnostic criteria for definite LNB, as outlined by the European Federation of Neurological Societies (EFNS), require the presence of neurological symptoms, cerebrospinal fluid (CSF) pleocytosis, and intrathecal production of *Borrelia*-specific antibodies.^7^

Diagnosing LB can be challenging, as the development of *Borrelia*-specific antibodies typically takes several weeks after the initial infection.^8–10^ During this period, diagnosis is primarily based on clinical findings. In disseminated LB cases, such as LNB, serology-based tests are generally effective; however, they struggle to distinguish between active infection and past LB episodes due to persistently elevated *Borrelia*-specific antibody levels, even after successful antibiotic treatment.^11^

Machine learning (ML) models are rapidly gaining popularity in microbiology, offering versatile tools to interpret vast and complex data by identifying patterns, uncovering relationships and supporting data-driven decision-making. ML applications have been used for e.g., virus–host sequence-based protein–protein interaction prediction,^12^ thyroid cancer,^13^ breast cancer,^14,15^ prostate cancer^16^, Parkinsons’s disease^17,18^ and COVID-19 diagnosis.^19,20^ Kehoe et al. (2022) conducted an important study on the use of sparse support vector machines (SSVM) for metabolite-based diagnostics for LB.^21^ Their findings underscore the diagnostic potential of ML-based metabolomics in LB and serve as a conceptual foundation for our current work, which extends this approach to LNB using serum samples from definite acute LNB patients.

In our previous study, we performed ultrahigh-performance liquid chromatography-tandem mass spectrometry (UHPLC-MS/MS)-based metabolomic profiling of serum samples from patients with definite acute LNB, collected both before and 12 months after antibiotic treatment.^22^ Building on these data,^23^ we developed ML-based classifiers trained on our data set, which also included age- and sex-matched non-LNB controls. A central feature of our study is its paired-sample design, enabling direct within-patient comparison of pre- and post-treatment metabolite profiles. To our knowledge, this study represents the first application of ML to paired serum metabolomics data in LNB, offering a novel opportunity to capture individual-level metabolomic changes associated with the disease.

## Results

### Metabolomic Data Characteristics

*In silico* metabolomic analysis of the sera identified 32,768 molecular features (MFs) from the UHPLC-MS/MS data. Of these, 27,021 MFs were assigned predicted molecular formulae, while 6,675 MFs received preliminary identifications. Furthermore, 1,606 identified MFs were linked to predicted metabolomic pathway associations. The median retention time (RT) of the MFs was 4.31 min within the MS data acquisition window, with a median mass-to-charge ratio (*m*/*z*) of 487.27896. The ML model’s top-200 MFs and their integrated peak areas are listed in Supporting Information Table S1. A subset of detected MFs has been characterized in our previous publication.^22^

### Supervised Machine Learning Model

To explore the diagnostic utility of the ML model, we developed supervised classifiers using MS-based serum metabolomics data. Training of the ML model was performed using sera from three clinically defined patient groups: patients with acute definite LNB (pretreatment), patients who had completed successful antibiotic treatment for LNB (post-treatment), and non-LNB controls that were age- and sex-matched for the LNB patients. It is important to note that the non-LNB control group includes individuals with medical conditions (infections, inflammatory conditions, autoimmunity, etc.) for which LB/LNB was considered as a possible diagnosis, based on the requested LB serology. By analyzing patterns in the detected small-molecule metabolites present in these samples, the ML model aimed to identify metabolic signatures associated with disease status and treatment response. This approach may support the development of more precise and data-driven complementary diagnostic tools for LNB.

Distance-based redundancy analysis (dbRDA) was performed on Euclidean distances, constraining the ordination by the three-level group variable after excluding observations with missing values. Permutational multivariate analysis of variance (PERMANOVA) restricted to the top-100 features (ranked by coefficient of variation) attributed 4% of the variance to group differences. Multivariate dispersion did not differ among groups (β-dispersion *p* = 0.37), indicating homogeneous within-group variability. Corresponding dbRDA plots are presented in Figure 1.

**Figure 1:**
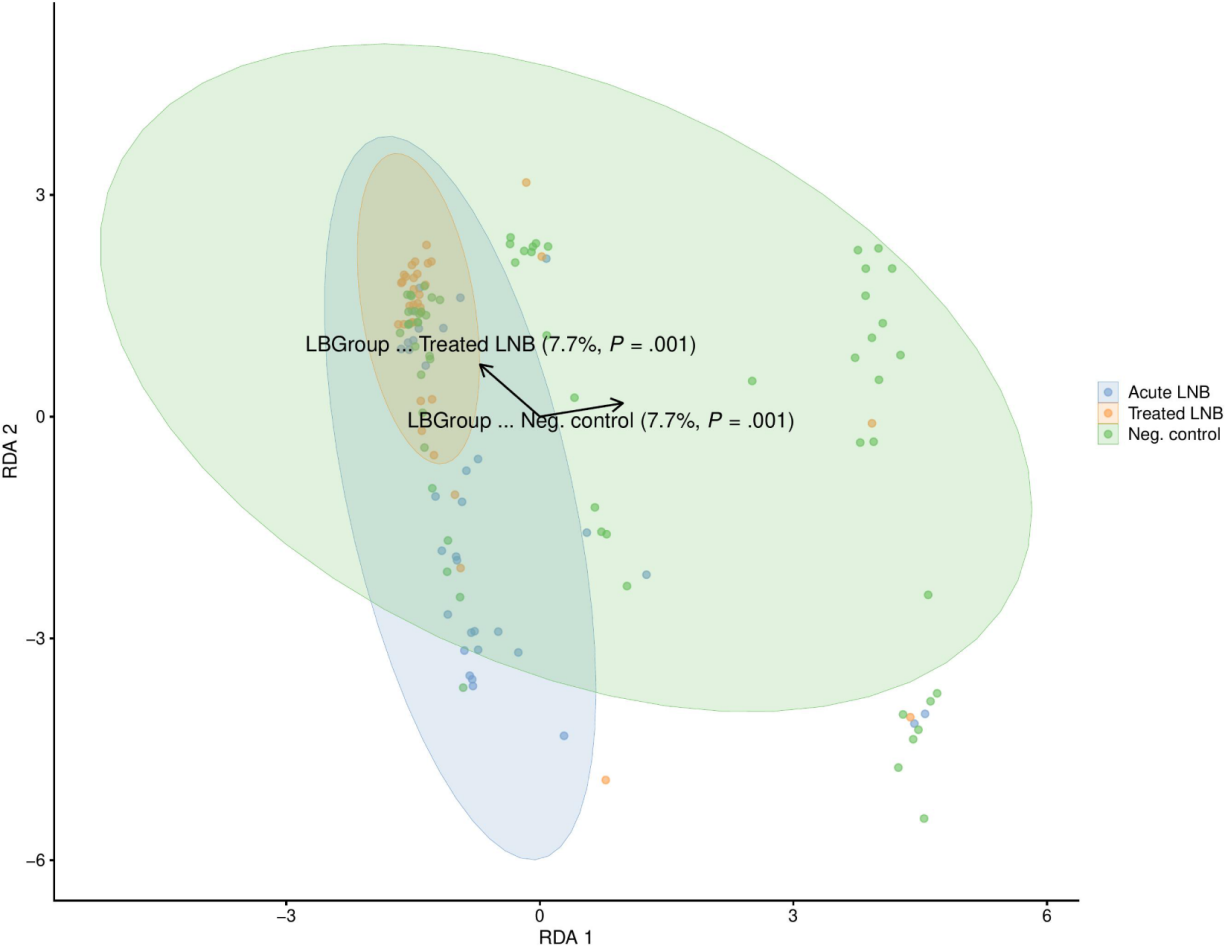
Distance-based redundancy analysis (dbRDA) of serum samples from Lyme neuroborreliosis (LNB) comparison groups: acute pretreatment LNB, treated LNB, and *Borrelia* antibody-negative (non-LNB) controls. The dbRDA visualization is based on the top-100 features with the highest coefficient of variation.

The original metabolomics data set, comprising 32,767 MFs, was reduced to 3,000 MFs with the highest CoV (SD/mean). These were further filtered to identify the top-200 features showing the most significant association with sample groups, based on a nonparametric Kruskal–Wallis test in the training data. The features belonging to the top-200, as well as the group-specific top-100 features are presented in Figure 2 and Random Forest importance score histograms for the top-200 features in Figure 3.

**Figure 2:**
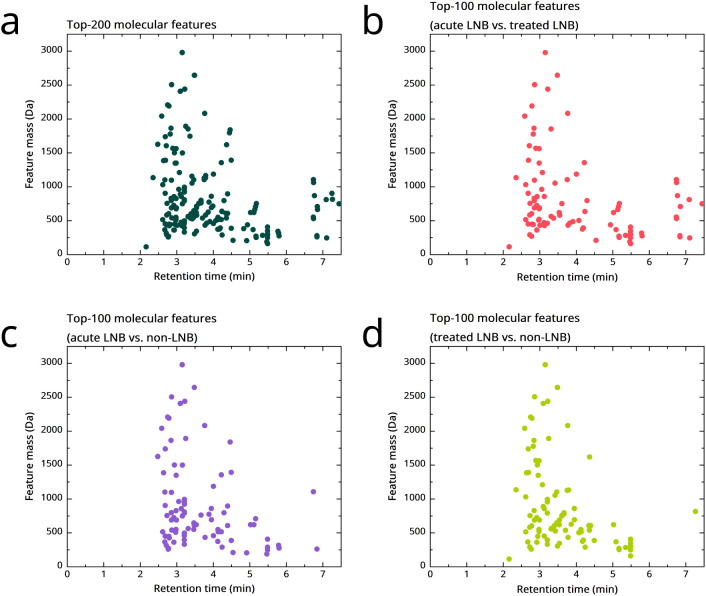
Top discriminative features identified from the ultrahigh-performance liquid chromatography-tandem mass spectrometry (UHPLC-MS/MS)-based metabolomic analysis. Retention time vs feature mass for the (**a**) top-200 features showing the most significant association with sample groups in the training set, based on the nonparametric Kruskal–Wallis test. Top-100 features identified from pairwise group comparisons: (**b**) acute pretreatment Lyme neuroborreliosis (LNB) vs post-treatment (treated) LNB, (**c**) acute pretreatment LNB vs *Borrelia* antibody-negative (non-LNB) controls, and (**d**) treated LNB vs non-LNB controls. Features are presented based on their characteristic exact masses and retention times as determined by UHPLC-MS/MS analysis.

**Figure 3:**
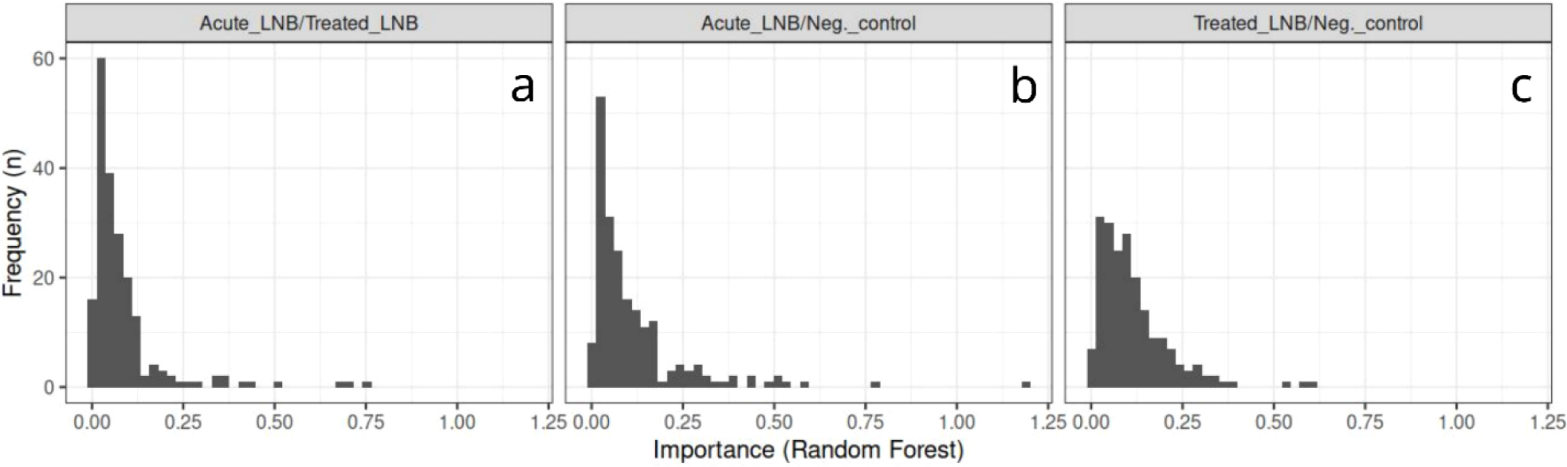
Random Forest importance score histograms for the top-200 molecular features in three different classifiers: **a**) acute Lyme neuroborreliosis (LNB) vs treated LNB; **b**) acute LNB vs Borrelia antibody-negative (non-LNB) controls; **c**) treated LNB vs non-LNB.

Among the top-100 discriminatory features identified in each pairwise comparison, a substantial number were shared across all three group comparisons, suggesting the presence of a core set of metabolites consistently altered in relation to LNB. Total of 22 MFs were shared by all three classifiers, indicating a cross-comparison signature that persists across both the longitudinal and case-control contrasts. Forty-four MFs overlapped between comparison **i** (acute LNB vs treated LNB) and comparison **ii** (acute LNB vs non-LNB controls), 50 MFs between **i** and comparison **iii** (acute LNB vs non-LNB controls), 47 MFs between **ii** and **iii** (each pairwise overlap includes the 22 MFs shared by all three comparisons). At the same time, each comparison retained a distinct subset of features unique to its top-100 list: 28 MFs unique to **i**, 31 MFs unique to **ii**, and 25 MFs unique to **iii**.

### LNB Classification

Three pairwise comparisons were conducted to evaluate the ML model’s ability to differentiate between the groups. The supervised ML model was first trained on a teaching set comprising 2/3 of the serum metabolomic profiles from the three patient groups. Model performance was then evaluated using an independent validation set consisting of the remaining 1/3 of the serum profiles, to ensure generalizability and minimize overfitting.

Comparison **i**, yielded an expected accuracy of 0.61 and an observed value of 0.80. Comparison **ii** showed an expected accuracy of 0.58 and an observed value of 0.86. Comparison **iii**, produced an expected accuracy of 0.63 and an observed value of 1.00. The expected accuracy value denotes the predicted level of group discrimination under baseline conditions, whereas the observed value reflects the model’s actual performance based on the data. The expected outcome depends also on the balance between the group sizes, for example an expected value of 0.5 would occur in a two-group comparison with equal group sizes. In our case, the group sizes were unbalanced, which affected the expected baseline. Moreover, the classification process includes stochasticity due to partial overlapping of class boundaries. Therefore, we present the naive baseline corresponding to random classification as a reference point. Based on these results, the Random Forest classifier clearly outperformed random classification and also revealed performance differences across the two-group comparisons, demonstrating desirable and robust classifier performance. These results are presented in Figure 4.

**Figure 4:**
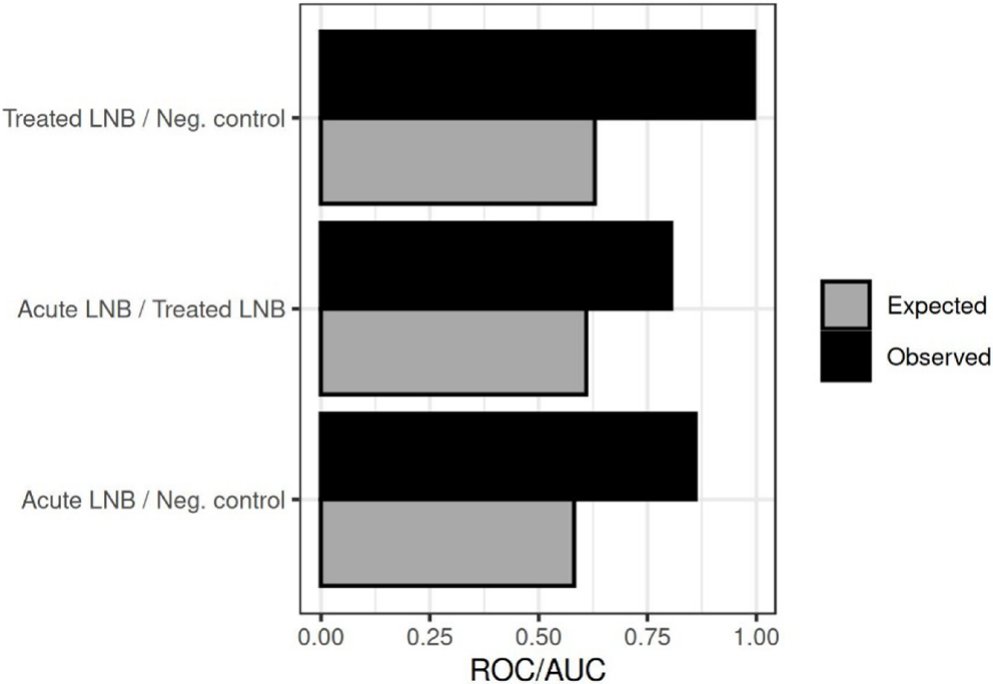
Receiver operating characteristic area under the curve (ROC/AUC) of the assessed three comparison groups.

To explore overall sample similarity across the different comparison groups, all serum metabolomic profiles were log_10_-transformed and feature-scaled prior to ordination. This preprocessing step reduces the influence of skewed distributions and ensures comparability across features. Ordination was performed on the entire data set without separating training and validation sets, allowing for a comprehensive overview of the relationships among all samples. The resulting representative plots in Figure 5 reveal clustering patterns that reflect underlying metabolic similarities and differences between acute pretreatment LNB, treated LNB, and non-LNB controls.

**Figure 5:**
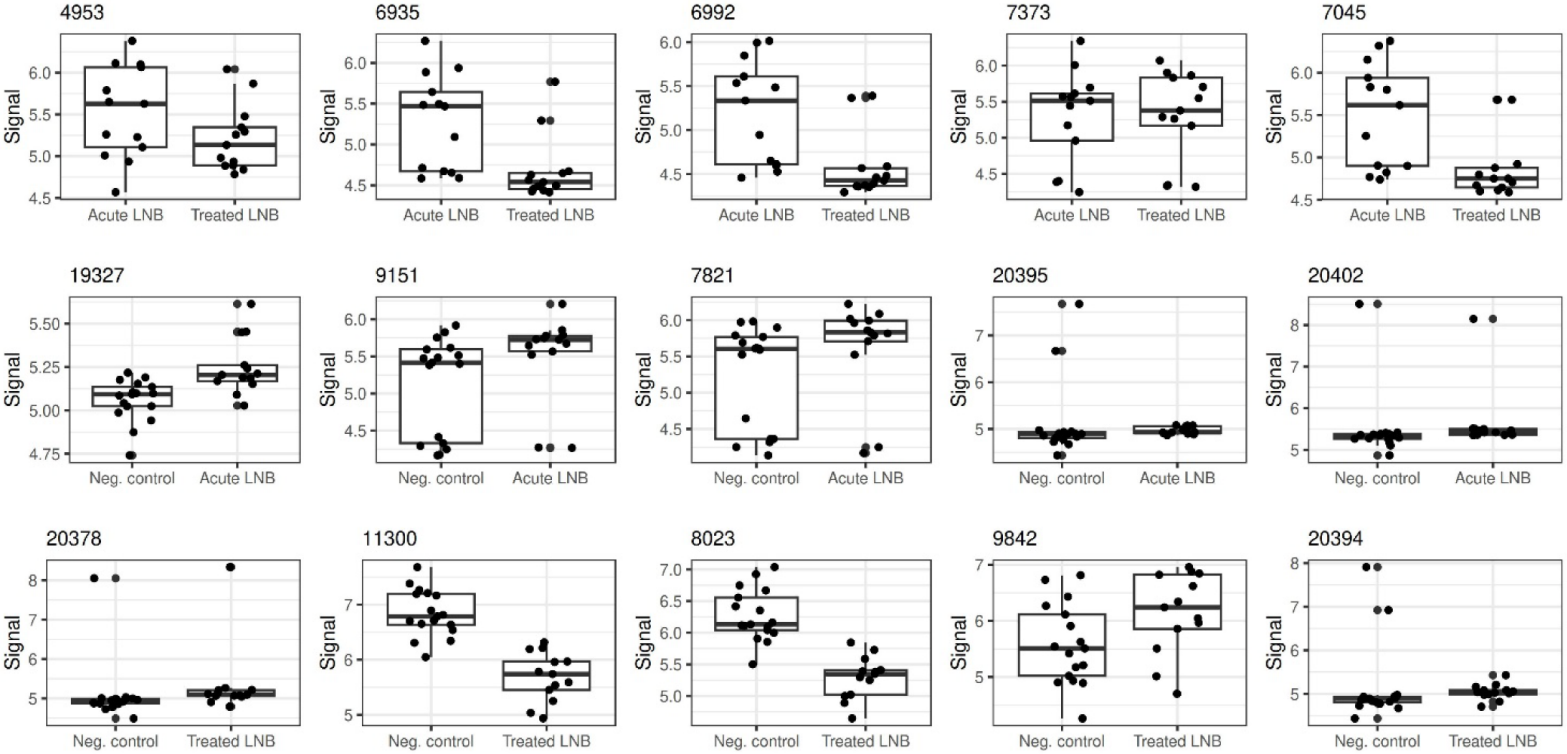
Boxplots of representative discriminatory metabolite signals for each pairwise comparison: acute pretreatment Lyme neuroborreliosis (LNB) vs treated LNB, acute pretreatment LNB vs *Borrelia* antibody-negative (non-LNB) controls, and treated LNB vs non-LNB controls. The selected features illustrate group-level differences in signal intensity. Values are log_10_-transformed and scaled. Each dot represents an individual serum sample.

## Discussion

This study demonstrates the potential of MS-based metabolomics combined with supervised ML to distinguish between definite acute pretreatment LNB, treated LNB, and non-LNB controls based on serum metabolic profiles. These findings are consistent with previous research, highlighting the diagnostic value of metabolomics in LB diagnostic research. Molins et al. developed LC-MS-based biosignatures that outperformed conventional serology in early LB diagnostics, effectively distinguishing the manifestation, not only from healthy individuals, but also from similar conditions such as Southern tick-associated rash illness (STARI).^23,24^ Fitzgerald et al. identified numerous metabolites altered in early LB, providing insights into host response mechanisms to *Borrelia* infection.^25^ Similarly, Kehoe et al. developed an effective ML model for early LB diagnostics using metabolomics data, achieving high test balance success rate of 98.13%.^21^ The metabolomic data was derived from Fitzgerald et al. (2020) who performed LC-MS analysis on serum samples from early state LB patients and healthy controls.^25^ The LB samples included cases of early localized LB, patients with a single visible EM rash and negative blood culture for *Borrelia*, and early disseminated LB, defined by multiple EM lesions, or by a single EM lesion and positive blood culture for *Borrelia*. Healthy controls were blood donors without a history of LB from both LB-endemic and nonendemic regions; these controls were not age- or sex-matched to the early LB samples.^25^ In the context of LNB, our previous work using UHPLC-MS/MS identified 53 candidate serum biomarkers capable of differentiating acute pretreatment LNB from post-treatment profiles.^22^ Collectively, these studies support our current findings, reinforcing that metabolomics can capture stage-specific molecular signatures, including persistent post-treatment alterations, even in the absence of clinical symptoms.

The dbRDA analysis shows that pretreatment LNB patient group vs other groups accounted for 7.7% of the total variation in the data. Whereas dbRDA explains only a moderate proportion of the overall community variation, the Random Forest ML classifier detects subtle but systematic differences between the groups that can be used for accurate classification despite their moderate contribution to the overall variation in the data.

The supervised ML model performed significantly better than expected by chance, across all three group comparisons. In comparison **i**, the observed accuracy rate was 0.80, exceeding the expected 0.61. This supports the notion that treatment induces detectable metabolic changes, although the remaining overlap between groups may reflect interindividual variation in recovery dynamics. The discrepancy between observed and expected ID values should, however, be interpreted with caution. Beyond model calibration, such gaps can be due to complex metabolomic data sets and may arise from a combination of biological heterogeneity, technical variation in MS, and preprocessing effects.^26–28^ In comparison **ii**, the observed accuracy was 0.86, clearly surpassing the expected 0.58, suggesting strong metabolomic differentiation between active disease and non-LNB conditions. The most striking finding, however, was in comparison **iii**, where a perfect accuracy of 1.00 was observed (compared to an expected value of 0.63).

These results were further supported by ROC curve analyses, which consistently demonstrated robust discriminatory performance across all comparisons. This suggests that even after apparent clinical recovery, treated LNB patients retain a distinct metabolic signature relative to non-LNB individuals. The perfect separation between treated LNB and non-LNB controls may even hold practical value, for example in identifying individuals with prior *Borrelia* exposure or contributing to seroprevalence estimation in diagnostically uncertain cases. At the same time, this raises important questions about the long-term biological consequences of infection and the effects of treatment. It should also be acknowledged that, while the non-LNB controls were confirmed *Borrelia*-negative, other infections or immune-related conditions cannot be fully excluded and may have contributed to the observed group separation.

A central observation of this study is the consistency of discriminative MFs across clinically distinct comparisons **i**–**iii**, pointing to a core, infection-related metabolic signature associated with LNB. Among the top-100 features identified for each pairwise classifier, 22 MFs were shared by all three. This cross-comparison overlap indicates that the same metabolites discriminate acute infection from recovery and LNB patients (both acute and treated) from non-LNB controls, supporting the notion that these features are linked to infection biology rather than to a single treatment state. Pairwise MF overlapping further underscore this continuity: 44 MFs overlapped between **i** and **ii**, 50 MFs between **i** and **iii**, and 47 MFs between **ii** and **iii**. Each of these counts includes the 22 MFs common to all three comparisons. In parallel, each comparison retained a distinct subset of unique features, 28 (**i**), 31 (**ii**), and 25 (**iii**), referring to comparison-specific biology (e.g., convalescent recovery) layered on top of the shared infection-linked core. Within each classifier, the majority of features also overlapped with the other two (**i**: 72, **ii**: 69, **iii**: 75), again consistent with a pervasive, disease-related signal.

Several mechanisms may contribute to the persistent metabolic differences observed between treated LNB patients and non-LNB controls, even well after clinical recovery. One contributing factor is the continued elevation of *Borrelia*-specific antibodies, which, although gradually declining, can persist for months after treatment.^11,29^ Our findings are also consistent with those of Clarke et al. (2021), who reported that peripheral blood mononuclear cell (PBMC) transcriptomic profiles in LB patients remained distinct from healthy controls even one year after treatment.^30^ Their work showed persistent upregulation of immune-related genes, suggesting that the immune system retains a long-term activation signature despite apparent clinical resolution. This persistent gene expression profile may have direct metabolic consequences, helping to explain why serum metabolomic profiles in treated LNB patients continue to diverge from those of seronegative controls.

One factor to consider is the known impact of antibiotic treatment on the host microbiome. Broad-spectrum antibiotics, such as the used doxycycline and ceftriaxone, can influence the microbiome and systemic metabolome and may contribute to long-term metabolic variation.^31,32^ Jaskiw et al. (2021) demonstrated that broad-spectrum antibiotic treatment reduced serum concentrations of key gut microbiota dependent metabolites, including *p*-cresol sulfate, phenol sulfate, hippurate and indole derivatives, indicating suppression of gut microbial activity.^33^ Although our second sampling occurred 12 months after antibiotic treatment, studies suggest that antibiotic-induced perturbations of the gut microbiome and associated metabolites may persist well beyond the acute treatment phase.^34,35^ In-feed antibiotic exposure in animals was shown to significantly alter systemic amino-acid metabolism and biogenic amines.^35^ Ma et al. (2023) demonstrated that antibiotics can induce profound short-term disruptions of gut microbial communities and systemic metabolic networks, particularly in amino-acid and bile-acid pathways.^36^ Despite these findings, few studies have examined the long-term metabolic consequences of antibiotic exposure in large-scale human cohorts, leaving the extent of microbiome recovery and metabolic adaptation largely unexplored. Importantly, antibiotic exposure occurred only in the treated LNB group. Acute LNB samples were collected prior to treatment initiation, and the non-LNB controls did not receive antibiotics as part of the study. This design feature helps interpret treatment effects from infection-related biology.

This interpretation is further strengthened by the performance of the supervised models across all contrasts, including acute pretreatment LNB vs non-LNB (ML model accuracy 1.00) and by the persistence of discriminative features 12 months after treatment. The presence of the same core features in the pretreatment vs non-LNB comparison indicates that these metabolites are not merely artifacts of antibiotic exposure. Instead, they are consistent with a disease-related metabolic fingerprint that spans the acute and post-treatment phases. However, this does not completely rule out a contributory role for antibiotics or other exposures. Instead, the fact that same core features were observed across all comparisons, and especially that they were present before treatment, supports infection as the primary driver.

At the same time, the unique feature sets per comparison are biologically informative. These features likely capture state-dependent aspects, such as acute inflammation, immune activation, or convalescent recovery, that distinguish acute pre- and post-treatment states or differentiate LNB from non-LNB at specific time points. Such comparison-specific signals may reflect heterogeneity in disease severity, recovery linked alternations, or host factors (e.g., microbiome composition). Beyond biological variation, technical factors and preprocessing choices inherent to untargeted metabolomics can also influence feature selection; thus, these unique sets should be viewed as candidates for targeted validation rather than definitive biomarkers at this stage.

Taken together, our data support a two-tier interpretation: (**1**) a shared, infection-linked core of MFs (the 22 MFs present in all three comparisons) that likely represent stable, disease-related signals and merit highest priority for targeted assay development and external validation; (**2**) comparison-specific MFs that may be clinically relevant state information (e.g., acute vs. recovery) and need follow-up studies to clarify mechanism and translational utility.

Together, these findings support the idea that post-treatment LB leaves a durable immuno-metabolic fingerprint, driven by persistent immune activity.^30^ Rather than representing residual noise, the metabolic differences identified through our UHPLC-MS/MS analysis and ML pipeline reflect biologically meaningful systemic alterations. The current applicability of the ML model is primarily research focused. The model highlights candidate MFs that can group patients into clinically relevant subtypes, capture recovery over time, and guide target MF selection for validation. Clinical use appears promising as an adjunct, supporting recovery monitoring and aiding differential diagnosis when interpreted alongside clinical findings. Standalone diagnosis, treatment decisions, or screening are not supported at this stage. Advancing applicability will require external, multicenter, longitudinal validation, harmonized preanalytical and analytical procedures, defined reference ranges and decision thresholds, and comparisons against existing diagnostic and monitoring tools for LB/LNB.

A major strength of this study is the use of paired serum samples from the same individuals before and after treatment. This within-patient design minimizes interindividual variability and increases sensitivity to biologically meaningful, patient recovery-related changes. In addition, the non-LNB control group was age- and sex-matched to the LNB cases, helping to control for demographic confounders. Although the non-LNB sera were not obtained from completely healthy individuals, their inclusion also remains valuable for distinguishing general infection or inflammation-related markers. In contrast, the treated LNB patients, who had clinically recovered at the time of sampling, served as a biologically relevant reference group. This group approximates a postinfectious but otherwise healthy state, providing a meaningful baseline for interpreting long-term metabolic changes following LNB.

Despite these promising results, several limitations should be acknowledged. The variance explained by group differences in unsupervised ordination was relatively low (PERMANOVA 4%), although it was statistically significant. This indicates that while group-level separation exists, it accounts for only a portion of the total variance, reflecting the complexity and heterogeneity of the metabolic data. The sample size was limited, and no independent external test cohort was used for validation, which restricts the generalizability of the findings. Moreover, although the non-LNB group was matched and clinically relevant, the inclusion of healthy controls would strengthen baseline comparisons. Finally, descriptive data on the frequency of comorbidities or detailed medication use was not available due to restrictions in the study permit for the samples. This may represent an additional source of variation that could not be fully accounted for in the present analysis.

Future work should focus on expanding sample sizes, including broader clinical phenotypes (different LB manifestations and other infections) and recruitment of healthy individuals alongside antibiotic-only subjects. Rigorous validation in independent cohorts and multitime point sampling will be essential to identify infection-related effects from those of treatment and other exposures. In addition, integrating other omics layers, such as transcriptomics, could further enhance the biological interpretability and clinical utility of the findings.

## Conclusions

Our study shows that serum metabolomics, when applied in a carefully designed longitudinal study, can effectively capture both disease and recovery-related metabolic signatures in LNB. The ability to accurately distinguish treated LNB patients from non-LNB controls, achieving a perfect identification rate, highlights a potential role for ML and metabolomics in identifying patients with previous infection episodes. This approach may also be valuable in epidemiological studies of seroprevalence. Within-patient sampling provides a powerful design advantage, and the findings offer a compelling case for further development of metabolomics-based complementary tools in the diagnosis and monitoring of LNB.

## Experimental Section

### Serum Samples of LNB and Non-LNB Patients

Pre- and post-treatment serum samples from patients with definite acute LNB were collected from a total of 34 individuals during routine LB diagnostics, as part of our previous study.^37^ These patients were treated either with intravenous ceftriaxone or oral doxycycline. Non-LNB serum samples were obtained during routine serology diagnostics from 62 individuals who were suspected of having LNB but showed no detectable intrathecal *Borrelia*-specific antibody production and were also seronegative for *Borrelia* using an in-house whole-cell antigen preparation-based enzyme immunoassay test. All serum samples were stored at −20 °C, which is a standard storage temperature for routine diagnostic samples. No freezers with automated defrost cycles were used. Additional information on the LNB cohort is available in our previous publications.^22,37^

Informed consent was obtained from all participants in the original study, with ethical approval granted by the National Committee on Medical Research Ethics in Finland.^37^ Permission for the study was obtained from the Wellbeing services county of South-West Finland (T13_2019–1/381700). LNB diagnoses were confirmed following the guidelines and criteria established by the EFNS.^7^ The left-over non-LNB samples used as negative controls were identified retrospectively from the laboratory information management system of the Clinical microbiology laboratory of Turku University Hospital. The negative samples were collected with informed consent from patients suspected to have LB/LNB as a part of routine clinical practice. All samples were coded, and strict anonymity was maintained throughout the study. This study was conducted in accordance with the ethical principles of the Declaration of Helsinki for research involving human subjects and biological data.

### UHPLC-MS/MS and the *In Silico* Metabolomic Data Analysis

The sample preparation process and UHPLC-MS/MS analysis have been described in detail in our previous publication.^22^ Serum samples from non-LNB control patients were analyzed alongside those from LNB patients using the same analytical platform and conditions, as reported previously.^22^

We performed *in silico* UHPLC-MS/MS metabolomic data analysis with Compound Discoverer 3.1 (Thermo Fisher Scientific Inc., Waltham, MA, USA; version 3.1.0.305). MF detection was conducted using a minimum peak intensity threshold of 500,000 (base peak height) and a maximum peak width of 0.5 min. A mass tolerance of 5 ppm was applied for MF grouping, elemental composition prediction, and library matching. For high-resolution fragment masses in mzCloud searches, an error tolerance of 10 ppm was applied, along with a relative intensity tolerance of 30% for isotope pattern matching. A signal-to-noise (S/N) threshold of 3 was used for centroid processing, and a minimum RT tolerance of 0.2 min was applied for feature grouping. Additional details regarding the *in silico* metabolomic analysis parameters are provided in our previous publication.^22^

### Machine Learning Model

We imported data in the TreeSummarizedExperiment data structure in R/Bioconductor.^38,39^ A total of 130 serum samples available for the ML model, comprising pretreatment LNB samples (n = 34), post-treatment LNB samples (n = 34), and non-LNB control samples (n = 62). These samples represented a total of 62 analytical data cases used for model development, comprising 34 complete matched triplets (pretreatment, post-treatment, and control) and additional 28 individual control instances. The data was split into training and test sets per individual in order to avoid data leakage and quantify model performance in a distinct set of patients that the trained model has not seen during the training phase. Two-thirds of the data cases (i.e., individuals; n = 41) were used for model training, while one-third (n = 21) of the data cases (individuals) were left out from model training in order to subsequently evaluate the model predictions. The signal was transformed with log_10_ before the analysis. We used the Random Forest algorithm as implemented in the mikropml R package.^40^ A separate classifier was trained between each pair of the three groups (acute/treated/control). We used 5-fold cross-validation within the training set, with an 80% training fraction. Model performance was evaluated by constructing confusion matrices that compared the predicted and known outcomes in the leave-out test data set based on the Random Forest classifier trained on the same features.^41^ Classification accuracy, sensitivity, specificity and receiver operating characteristic area under the curve (ROC/AUC) values were then calculated and compared to random predictions. Feature importance was derived from the Random Forest models as the mean Gini decrease value. This is a standard measure that ranks variables by how much they improve class separation across the trees, identifying those variables the classifier relies on most to distinguish the patient groups in the held-out test set.

## Supplementary Material



## Data Availability

The metabolomic data supporting the findings of this study are available in Supporting Information and the ML source code at https://www.doi.org/10.5281/zenodo.18956198.

## Notes

Table S1: machine learning model’s top-200 molecular features and their integrated peak areas obtained from Compound Discoverer 3 *in silico* metabolomic analysis of serum mass spectrometric data (XLSX)

## Abbreviations

AUC: Area under the curve
CNS: Central nervous system
CSF: Cerebrospinal fluid
CoV: Coefficient of variation
dbRDA: Distance-based redundancy analysis
EFNS: European Federation of Neurological Societies
EM: Erythema migrans
LB: Lyme borreliosis
LC: Liquid chromatography
LNB: Lyme neuroborreliosis
*m*/*z*: Mass-to-charge ratio
MF: Molecular feature
ML: Machine learning
MS: Mass spectrometry
MS/MS: Tandem mass spectrometry
PBMC: Peripheral blood mononuclear cell
PERMANOVA: Permutational multivariate analysis of variance
RT: Retention time
ROC: Receiver operating characteristic
SSVM: Sparse support vector machine
STARI: Southern tick-associated rash illness
S/N: Signal-to-noise ratio
UHPLC: Ultrahigh-performance liquid chromatography
